# Development and Validation of an Immune-Related lncRNA Signature for Predicting the Prognosis of Hepatocellular Carcinoma

**DOI:** 10.3389/fgene.2020.01037

**Published:** 2020-09-04

**Authors:** Weihao Kong, Xingyu Wang, Xiaomin Zuo, Zhongxiang Mao, Ya Cheng, Wei Chen

**Affiliations:** ^1^Department of Emergency Surgery, The First Affiliated Hospital of Anhui Medical University, Hefei, China; ^2^Department of Emergency Medicine, The First Affiliated Hospital of Anhui Medical University, Hefei, China; ^3^Department of General Surgery, The First Affiliated Hospital of Anhui Medical University, Hefei, China

**Keywords:** immune, lncRNA, signature, prognosis, nomogram, hepatocellular carcinoma

## Abstract

**Aim:**

Immunotherapy is currently being explored as a potential treatment for hepatocellular carcinoma (HCC). This study investigated the prognostic value of immune-related long non-coding RNAs (lncRNAs) in patients with HCC.

**Methods:**

The Wilcoxon test was used to compare differentially expressed lncRNAs between HCC tissue and non-tumor tissue. Moreover, co-expression analysis was used to determine immune-related lncRNA. Univariate cox regression analysis and the least absolute shrinkage and selection operator (LASSO) regression were used to identify immune-related prognostic lncRNA. The immune risk score was calculated by the sum of the product from each lncRNA expression and its coefficient. Furthermore, the prognostic significance of the lncRNA signature was determined in the training group, testing group, and the entire group. A prognostic nomogram was established by integrating immune risk score and clinicopathological features.

**Results:**

PRRT3-AS1 and AL031985.3 were identified as immune-related prognostic lncRNAs in HCC patients. HCC patients were divided into high and low-risk groups based on the optimal cutoff value of risk score in the training group. The prognosis of HCC patients in the high-risk group was worse compared with the low-risk group. Besides, the immune-related lncRNA score was regarded as an independent risk factor for the prognosis of HCC patients. The predictive nomogram showed satisfactory discrimination and consistency. Gene enrichment analysis results indicated that the high-risk group was associated with immune-related signaling pathways.

**Conclusion:**

This study screened a 2-lncRNA signature and constructed a nomogram to predict the survival of HCC patients, thereby provided guidelines for undertaking medical decisions.

## Introduction

Hepatocellular carcinoma (HCC) is one of the most prevalent cancers globally. It is highly aggressive and is characterized by early distant metastasis thus liver cancer patients exhibit a high recurrence rate and short survival time. Despite the rapid development of surgical methods such as surgery, chemotherapy, and radiation therapy, the 5-year overall survival rate of patients with hepatocellular carcinoma is very low (Chen et al., 2016; Yang et al., 2019). Therefore, there is an urgent need to identify useful molecular markers to predict the prognosis of patients with liver cancer. Recent research indicates that abnormal immune response in the tumor microenvironment plays a vital role in the occurrence and progression of various cancers such a liver cancer (Brown et al., 2019; Denaro et al., 2019). A study by Liu described that natural killer (NK) cells play an essential part in innate and adaptive immune defenses of the body. The impaired regulatory function of NK cells will alter the ability of the body to eliminate tumor cells thereby promoting tumor occurrence and progression (Liu et al., 2018). Besides, Yu reported that overexpression of lncRNA FENDRR can prevent the immune escape response of Treg-mediated liver cancer cells (Yu et al., 2019). Therefore, identifying targets associated with the immunity against tumors is significant in cancer treatment.

Long non-coding RNAs (lncRNAs) are RNA molecules composed of more than 200 nucleotides in length. They critically function in regulating chromatin, gene expression, growth, differentiation, and development (Necsulea et al., 2014; Jain et al., 2017). Besides, lncRNAs have an indispensable role in regulating T cells, B cells, dendritic cells, bone marrow-derived stem cells, tumor microenvironment, epithelial-mesenchymal transition, and microbial population balance (Atianand et al., 2017; Denaro et al., 2019). A study by Denaro reported that T cell activity plays an essential role in the tumor microenvironment and associated with prognostic outcomes of various tumors (Denaro et al., 2019). Therefore, the search for immune-related lncRNA in liver cancer may provide a new target for HCC treatment. Current researches have explored many lncRNA signatures associated with tumor prognosis (Gu et al., 2018; Zhao et al., 2018; Zhou et al., 2019; Li et al., 2020; Shen et al., 2020; Zhang et al., 2020; Zheng et al., 2020). However, fewer lncRNA signatures have been identified in liver cancer since many studies focus on microRNAs and mRNAs. This study, therefore, purposed to establish an immune-related lncRNA signature of liver cancer, and provide strategies for treating HCC patients.

## Materials and Methods

### Data and Patients

The RNA sequencing data (FPKM) for 374 liver cancer and 50 non-tumor tissues were downloaded from the TCGA website^1^. The clinicopathological characteristics of the corresponding liver cancer patients were downloaded from the cBioPortal website^2^ (Cerami et al., 2012). Information on lncRNA expression was extracted from the mRNA expression profile data based on the GTF file information downloaded from the GENECODE website^3^.

### Identifying the Differentially Expressed lncRNAs

The Wilcoxon test was used to explore differentially expressed lncRNAs between liver cancer tissue and normal tissue. Notably, *P*-value < 0.05 and | log2(fold change)| > 1 were used as screening criteria for differentially expressed lncRNAs. In cases with multiple identical lncRNAs, the average of the expression levels was obtained. The identified differentially expressed lncRNAs and their expression matrix was used for subsequent analysis.

### Immune-Related lncRNAs

Immune-related genes were downloaded from the GSEA molecular signature database v7.0, which included the immune response M19817 and the immune system process M13664^4^. Co-expression analysis was conducted between lncRNAs and immune-related genes. LncRNA with a correlation coefficient of more than 0.5 and a *P*-value of less than 0.01 were considered as immune-related lncRNAs.

### Establishing an Immune-Related lncRNA Signature

The following screening criteria were used to construct an immune-related lncRNA prediction model. The inclusion standard for this study included patients with complete prognostic information and pathologically confirmed hepatocellular carcinoma. The exclusion criterion was patients with less than 30 days of survival because they were more likely to die from non-tumor factors such as post-operative bleeding or infection. A total of 342 liver cancer patients were enrolled in this study. They were randomly divided into training (*n* = 171) and testing groups (*n* = 171) (Table 1). The univariate Cox regression model was utilized to screen for lncRNAs that were significantly associated with the overall survival (OS) of liver cancer in the training group (*P* < 0.01). The survival-associated lncRNAs were incorporated into the least absolute shrinkage and selection operator (LASSO) model. The optimal penalty parameter is determined by 10-fold cross-validation using the R package (glmnet) to prevent the model from overfitting. Finally, immune-related prognostic lncRNAs were obtained to construct an immune-related lncRNA risk score formula. Of note, the risk score formula = the sum of the product of the regression coefficient of lncRNAs and its corresponding expression. Based on the ROC curve of a risk score for predicting overall survival, we determined the optimal cutoff value with the maximum Youden index in the training group and divided HCC patients into high and low-risk groups. The Kaplan–Meier method was performed to explore survival differences between high and low-risk groups. The same cutoff value was subsequently used for the testing group and the entire group.

**TABLE 1:** Clinical information of the 342 patients with liver cancer.

Variables	Number of patients *n* = 342	Training set *n* = 171	Testing set *n* = 171	*P*-value
Age (years)	61.0 (18.0)	62.0 (17.0)	61.0 (17.0)	0.604
Gender, male/female	233/109	119/52	114/57	0.562
AJCC Stage, I/II/III/IV/NA	161/77/80/3/21	77/45/36/2/11	84/32/44/1/10	0.451
Histologic grade, G4/G3/G2/G1/NA	53/161/111/12/5	27/79/58/5/2	26/82/53/7/3	0.934
Survival status, Alive/Dead	219/123	109/62	110/61	0.910
Survival time (months)	20.7 (25.8)	20.0 (24.6)	21.0 (27.0)	0.620

### Identifying the Independent Prognostic Parameters of Liver Cancer

To verify the prognostic role of immune-related lncRNA risk score and explore potential prognostic-related clinicopathological features, the parameters including age, gender, histologic grade, AJCC stage, and risk score were used to perform the univariate and multivariate cox regression analysis on the TCGA training group, testing group, and entire group. Significant variables in univariate Cox regression analysis were incorporated into multivariate regression analysis using the stepwise method.

### Nomogram Construction

The nomogram was constructed from statistically significant variables from multivariate regression analysis in the training group. The discrimination and calibration of the predictive nomogram were evaluated using the concordance index (C-index) and the calibration curve, respectively. Similarly, the time-dependent receiver operating characteristic (ROC) curve and the decision curve analysis (DCA) is used to compare the predictive significance of the clinicopathological features and nomogram.

### Gene Set Enrichment Analysis

Gene set enrichment analysis (GSEA)^5^ was used to analyze the biological function and pathway of immune-related lncRNA signature. The input of GSEA is Expression matrix dataset and phenotype labels. The expression matrix dataset contains genes, samples, and an expression value for each sample. The phenotype labels represent the grouping information of the sample. The background of GSEA operation is based on the JAVA program, and the gene set used for GSEA is curated gene sets (c2.cp.kegg.v6.2.symbols.gmt). Data were filtered using p-values less than 0.05 and FDR values less than 0.25.

### Statistical Analysis

Continuous variables that did not show a normal distribution were described by the median and interquartile range whereas, continuous variables showing normal distribution were described by the mean ± standard deviation (SD). Categorical variables were described by frequency. The Kruskal-Wallis test was used to compare the continuous variable. The chi-square analysis was used to compare categorical variables. Survival analysis, univariate, and multivariate Cox regression analysis were used to explore the prognostic value of immune lncRNA signature. All statistical analyses were performed using GraphPad Prism 7 (GraphPad Software Inc, La Jolla, CA, United States), SPSS 24.0 software (SPSS, IL, United States) and R software (version 3.5.1). A *P*-value of less than 0.05 was considered statistically significant.

## Results

### Differentially Expressed lncRNAs in Hepatocellular Carcinoma

**FIGURE 1:**
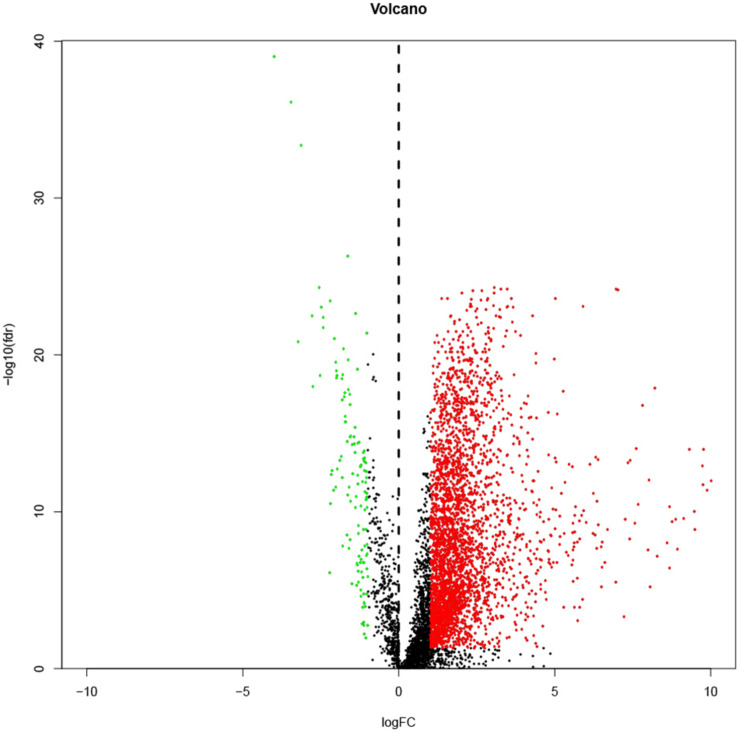
A volcano map showing all lncRNAs. Black dots represent insignificant lncRNAs in tumor tissues; green and red dots represent down-regulated and up-regulated lncRNAs in tumor tissues.

lncRNA expressions were extracted from 374 liver cancer samples and corresponding 50 normal samples from the TCGA database and subjected differential expression analysis. The accession IDs of liver cancer and normal samples are shown in Supplementary File S1. Following the previous criteria, a total of 3,405 (24.1%) differential lncRNAs including 126 (0.9%) down-regulated lncRNAs and 3279 (23.2%) up-regulated lncRNAs were extracted. A volcano map of all lncRNAs is shown in Figure 1. In the volcano plot, black dots represent insignificant lncRNAs in tumor tissues, green and red dots represent down-regulated and up-regulated lncRNAs in tumor tissues.

### Immune-Correlated lncRNAs in Hepatocellular Carcinoma

A total of 332 immune-related genes were obtained from the GSEA immune gene database. Co-expression analysis between the immune gene and lncRNA expression extracted from the TCGA HCC database generated 162 immune-related lncRNAs (| r| > 0.5, *P* < 0.01). The results of the correlation analysis of immune-related lncRNA and immune genes are shown in Supplementary File S2 (Correlation > 0.5, *P*-value < 0.01). Furthermore, 162 immune-related lncRNAs were used to construct a predictive lncRNA signature.

### Constructing and Validating an Immune-Related lncRNA Signature

**FIGURE 2:**
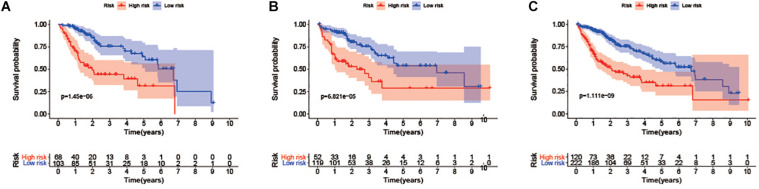
Survival curve analysis between high and low-risk groups in the training group: **(A)** Testing group. **(B)** The entire group **(C)**.

**FIGURE 3:**
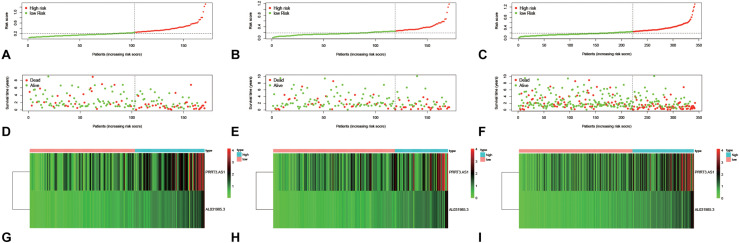
The distribution of immune risk score **(A–C)**, survival status **(D–F)**, and expression matrix **(G–I)** of the two lncRNAs for HCC patients in the training group **(A,D,G)**, testing group **(B,E,H)**, and the entire group **(C,F,I)**.

Following the inclusion criteria, 342 liver cancer patients were included to construct a predictive model and were randomly divided equally into training and testing groups. There were no statistically significant differences between the training group and the testing group in terms of age (*P* = 0.604), gender (*P* = 0.562), tumor stage (*P* = 0.451), histologic grade (*P* = 0.934), survival status (*P* = 0.910), and survival time (*P* = 0.620) (Table 1). The accession IDs of those included in the predictive models are shown in Supplementary File S3. Univariate Cox regression model was used to explore the correlation between the expression of immune lncRNAs and the prognosis of HCC patients in the training group whereby 12 prognosis-related lncRNAs were obtained (*P* < 0.01) (Supplementary Figure S1). The LASSO regression model was used to screen the immune-related lncRNAs that affect the prognosis (Supplementary Figure S2). Notably, two lncRNAs signature for HCC patients were identified in the training group (Table 2). The coefficients of these two lncRNAs were greater than zero and were considered risk factors for the prognosis of patients with liver cancer. Therefore, the formula: Immune-related lncRNA risk score = expression value of PRRT3-AS1^∗^0.032+ expression value of AL031985.3^∗^0.270 was established. Based on the optimal cutoff value (0.248) of the risk score in the training group, the HCC patients in the training group were divided into a high-risk group and a low-risk group. The survival analysis results indicated that HCC patients in the low-risk group had better survival outcomes compared with patients in the high-risk group (Log-rank *P*-value = 1.450e-06). The 3-and 5-year overall survival rates for HCC patients in the low-risk group were 75.7% and 64.0% whereas, the 3-and 5-year overall survival rates for HCC patients in the high-risk group were 44.1 and 31.4%, respectively (Figure 2A). Similarly, the distribution of immune risk score, survival status, and expression matrix of the two lncRNAs for HCC patients in the training group are shown in Figure 3. We performed validation on the testing group and the entire group to verify the prognostic role of the immune-related lncRNA signature. Of note, in the testing group, the 3-and 5-year overall survival rates of HCC patients in the low-risk group were significantly higher than those in the high-risk group (Log-rank *P*-value = 6.821e-05) (Figure 2B). Similarly, in the entire group, HCC patients in the high-risk group showed worse prognosis than patients in the low-risk group (Log-rank *P*-value = 1.111e-09) (Figure 2C). The distribution of immune risk score, survival status, and expression matrix of the two lncRNAs for HCC patients in the testing group and the entire group is shown in Figure 3.

**TABLE 2:** Two lncRNAs identified using the univariate Cox regression model and the LASSO regression model.

LncRNA	Log2FC	Hazard ratio of univariate Cox regression	*P*-value	Coefficient of LASSO regression model
PRRT3-AS1	1.982	1.112	0.00052	0.032
AL031985.3	1.724	1.813	3.5E-06	0.270

*lncRNA, Long non-coding RNA; Log2FC, log2(fold change); LASSO, Least absolute shrinkage and selection operator.*

### The Risk Score Is Independently Correlated With the Prognosis of HCC Patients

Univariate Cox regression analysis was performed on age, gender, tumor stage, histologic grade, and risk score to determine whether the immune-associated lncRNA risk score is an independent risk factor for the prognosis of patients with liver cancer. In the training group, the results from univariate Cox regression analysis indicated that the tumor stage (*P* = 0.001) and risk score (*P* < 0.001) were correlated with the prognosis of patients with liver cancer. Moreover, the tumor stage and risk score were incorporated into a multivariate regression analysis using the stepwise method. It was reported that the AJCC stage (*P* = 0.005) and risk score (*P* < 0.001) were independent risk factors for the prognosis of liver cancer patients in the training group (Table 3). Additionally, the results of the multivariate regression analysis showed that the tumor stage and risk score were independent risk factors for the prognosis of HCC patients in the testing group and the entire group (Tables 4, 5).

**TABLE 3:** Univariate and multivariate Cox regression analyses in the training group.

Variable	Univariate analysis			Multivariate analysis		
	*p*	Hazard ratio	95% Confidence interval	*p*	Hazard ratio	95% Confidence interval
Age (≥60/< 60)	0.694	1.116	0.647–1.925			
Sex (female/male)	0.854	0.948	0.539–1.669			
AJCC stage (IV+III/II+I)	**0.001**	**2.705**	**1.540–4.751**	**0.005**	**2.278**	**1.284–4.041**
Histologic grade (G4+G3/G2+G1)	0.874	1.045	0.606–1.803			
Risk score (high/low)	**< 0.001**	**3.301**	**1.883–5.787**	**< 0.001**	**2.942**	**1.671–5.179**

*Bold font means P < 0.05.*

**TABLE 4:** Univariate and multivariate Cox regression analyses in the testing group.

Variable	Univariate analysis			Multivariate analysis		
	*p*	Hazard ratio	95% Confidence interval	*p*	Hazard ratio	95% Confidence interval
Age (≥60/<60)	0.674	1.123	0.654–1.930			
Sex (female / male)	**0.030**	**1.833**	**1.059–3.171**			
AJCC stage (IV+III/II+I)	**< 0.001**	**3.241**	**1.885–5.572**	**<0.001**	**2.814**	**1.621–4.887**
Histologic grade (G4+G3/G2+G1)	0.674	1.127	0.645–1.969			
Risk score (high/low)	**< 0.001**	**2.822**	**1.637–4.863**	**0.002**	**2.352**	**1.354–4.084**

*Bold font means P < 0.05.*

**TABLE 5:** Univariate and multivariate Cox regression analyses in the entire group.

Variable	Univariate analysis			Multivariate analysis		
	*p*	Hazard ratio	95% Confidence interval	*p*	Hazard ratio	95% Confidence interval
Age (≥60/<60)	0.499	1.141	0.779–1.671			
Sex (female/male)	0.162	1.320	0.894–1.948			
AJCC stage (IV+III/II+I)	**< 0.001**	**2.835**	**1.934–4.157**	**< 0.001**	**2.465**	**1.672–3.636**
Histologic grade (G4+G3/G2+G1)	0.660	1.091	0.739–1.611			
Risk score (high/low)	**< 0.001**	**2.958**	**2.015–4.342**	**< 0.001**	**2.596**	**1.763–3.822**

*Bold font means P < 0.05.*

### Immune lncRNA Risk Score Correlates With HCC Progression

**FIGURE 4:**
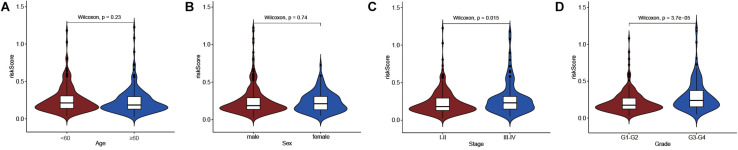
Differential analysis between risk score and clinicopathological features. **(A)** risk score and age. **(B)** risk score and sex. **(C)** risk score and AJCC stage. **(D)** risk score and Histologic grade.

We also explored the differences in immune-related lncRNA risk scores in different subgroups of clinicopathological characteristics. The results found that the risk score was significantly different in the AJCC stage (*P* = 0.015) and Histologic grade (*P* = 3.7e-05), but not for age (*P* = 0.23) and gender (*P* = 0.74) (Figure 4). A higher risk score was found in the inferior differentiation degree and advanced tumor stage.

### Gene Set Enrichment Analysis

**FIGURE 5:**
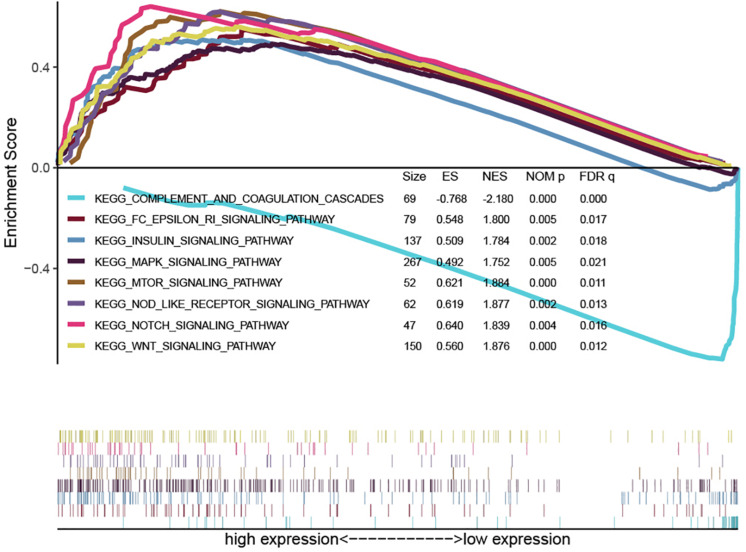
Representative results of the immune-related signaling pathway obtained by Gene Set Enrichment Analysis.

To explore the function of the 2-lncRNA signature, the entire group dataset was used for GSEA analysis. The GSEA results showed that the 2-lncRNA signature was significantly enriched in immune-related signaling pathways including mTOR signaling pathway, NOD-like receptor signaling pathway, MAPK signaling pathway, Notch signaling pathway, Wnt signaling pathway, Fc epsilon RI signaling pathway, Insulin signaling pathway, and complement and coagulation cascades (Figure 5). The enriched pathways of the high-risk group and the low-risk group by GSEA are shown in Supplementary Files S4, S5, respectively. Therefore, the 2-lncRNA signature may play a crucial role in tumorigenesis through these pathways.

### Nomogram Construction and Validation

**FIGURE 6:**
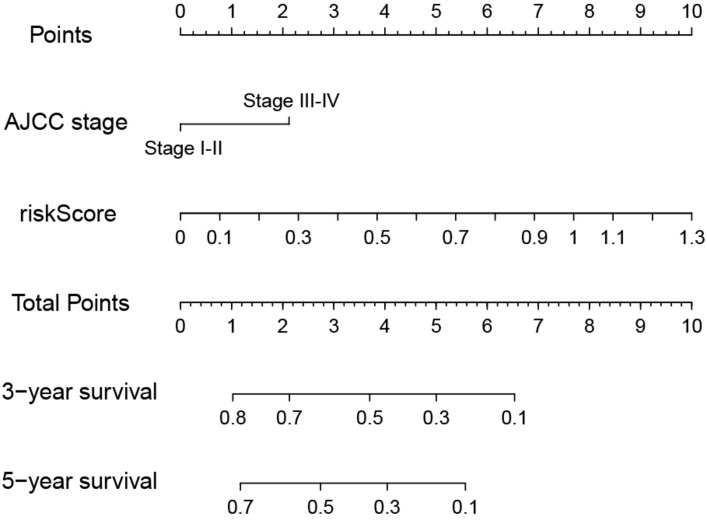
Predictive nomogram for OS in the training group. OS, Overall survival.

**FIGURE 7:**
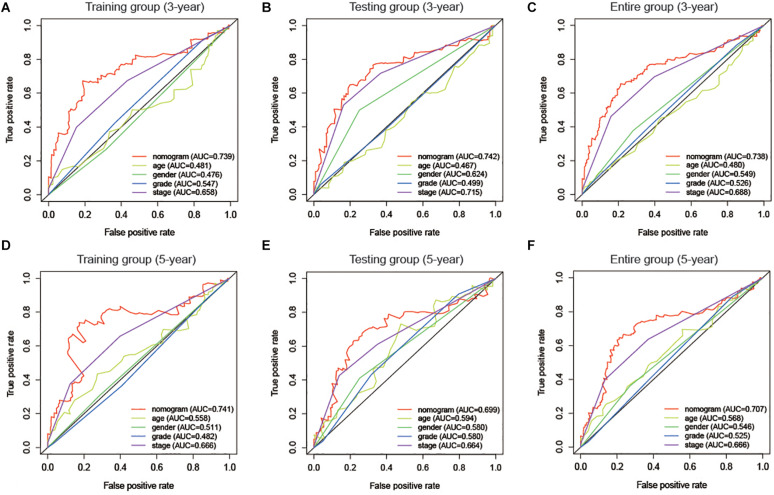
Time-dependent ROC curve analysis for the nomogram and the clinicopathological features in the training group **(A,D)**, testing group **(B,E)**, and the entire group **(C,F)**.

**FIGURE 8:**
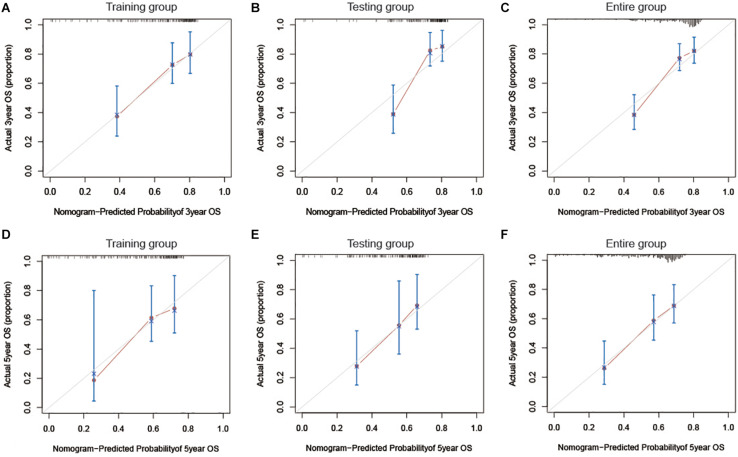
A 3-and 5-year calibration curve of OS in the training group **(A,D)**, testing group **(B,E)**, and the entire group **(C,F)**. OS, Overall survival.

**FIGURE 9:**
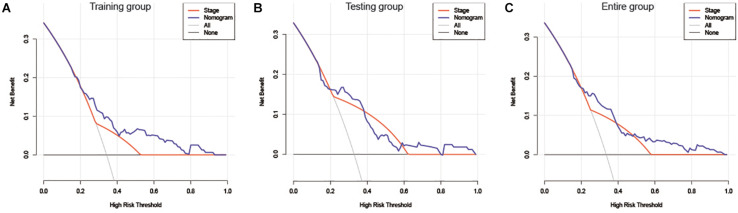
DCA of the nomogram and AJCC stage for OS in the training group **(A)**, testing group **(B)**, and the entire group **(C)**. DCA, decision curve analysis; AJCC stage, American Joint Committee on cancer; OS, overall survival.

The risk score and AJCC stage were reported as independent risk factors for the prognosis of patients with liver cancer according to the results of multivariate regression analysis in the training group. Therefore, an OS nomogram was constructed based on these two indicators (Figure 6). Based on the AJCC stage and the expression level of the two-lncRNA of HCC patients, we obtained the points of tumor stage and risk score in turn. We add up the two points to get the total points, and then make a vertical line to get the corresponding 3- and 5-year survival rates. For example, a liver cancer patient’s tumor stage is stage I, and its immune-related risk score is 0.51, then the patient’s tumor stage is 0 points, and the risk score is 3.93 points, and the total score is 3.93 points. Correspondingly, the patient’s 3-year survival rate and 5-year survival rate were 46.6 and 31.8%, respectively. A detailed example can be found in Supplementary Figure S3. Internal and external validation for the nomogram model was performed for the training group, testing group, and the entire group. The C-index in the internal validation group was 0.741 whereas, in the external validation group, the C-index for the testing group and the entire group was 0.676 and 0.707, respectively. Besides, the results of the ROC curve analysis showed that the nomogram model has better diagnostic value compared with the clinicopathological features in the training group, testing group, and the entire group (Figure 7). Furthermore, the results of the calibration curve showed that the 3-and 5-year survival rates predicted by the nomogram model presents the actual 3-and 5-year survival rates in the training group, the testing group, and the entire group (Figure 8). Finally, the results of the decision curve analysis show that the nomogram model has a better net benefit rate than the AJCC stage in the training group and the entire group. In the testing group, the net benefit rate of the nomogram model was similar to the AJCC stage (Figure 9).

## Discussion

In recent years, researchers can find biomarkers for tumor prognosis with the rapid advancement and popularization of chip sequencing technology. Many studies have reported that lncRNA plays an indispensable role in the invasion, migration, proliferation, apoptosis, and drug resistance of many tumors (Wang et al., 2019; Fan et al., 2020). Hence, identifying effective lncRNAs to predict the prognosis of cancer patients has gradually attracted various levels of scientific research. Moreover, researchers have explored many lncRNA predictive models to determine the prognosis of tumor patients including liver cancer, pancreatic cancer, lung cancer, esophageal cancer, colorectal cancer, breast cancer, among others (Gu et al., 2018, 2019; Zhao et al., 2018; Liu et al., 2019; Ma and Deng, 2019; Zhou et al., 2019; Shen et al., 2020; Zhang et al., 2020; Zheng et al., 2020). A study by Ma found that four lncRNA signature can effectively distinguish high-risk and low-risk patients in liver cirrhosis and liver cancer patients thereby accurately predict the prognosis of liver cancer patients (Ma and Deng, 2019). Moreover, his study confirmed that the immunity of the body plays a vital role in the occurrence and development of cancer. LncRNAs are critical factors involved in the immune response and play an essential role in tumor cell autophagy, immune response, and inflammatory response (Atianand et al., 2017; Denaro et al., 2019). Therefore, identifying immune-related tumor lncRNA can provide a new target for cancer treatment and strategies for clinical immunotherapy of cancer.

In this study, two immune-related lncRNAs were explored to predict the prognosis of HCC patients referring to the TCGA database. Moreover, the immune-related lncRNA risk score can effectively distinguish high-risk and low-risk patients in liver cancer. The results of the multivariate regression analysis also proved that it is an independent risk factor for the prognosis of HCC patients. Based on the results of multivariate regression analysis in the training group, we included the AJCC stage and immune-related lncRNA risk score to construct a nomogram model. The results of the C-index and the time-dependent ROC curve indicated that nomograms have satisfactory discrimination ability. The results of the calibration curves showed that the nomograms can accurately predict the prognosis of HCC patients. Finally, the results of the decision curve analysis show that the nomogram model has a better net benefit rate than the tumor stage. Therefore, the immune-related lncRNA signature is a potential biomarker for predicting the prognosis of HCC patients.

Moreover, a study by Fan explored the function of PRRT3-AS1 in prostate cancer and reported that PRRT3-AS1 silencing inhibited the invasion, migration, and proliferation of prostate cancer cells through the mTOR signaling pathway (Fan et al., 2020). However, no related research has reported the role of AL031985.3 in tumors, thus requires subsequent experiments. Notably, this study reported that immune-related lncRNA signature is significantly enriched in various immune-related signaling pathways, including the mTOR signaling pathway, NOD-like receptor signaling pathway, MAPK signaling pathway, Notch signaling pathway, among others. This is consistent with a report by Fun. Therefore, it may play an essential role in tumorigenesis and development through these pathways.

The lncRNA prediction models for liver cancer in this study were effective compared with other models from previous studies. First, we explored the immune-related lncRNA prediction model of liver cancer and provided potential therapeutic targets for targeted treatment of liver cancer. Second, based on the Cox regression model and Lasso regression model, two lncRNAs, PRRT3-AS1 and AL031985.3, were identified as risk factors for the prognosis of liver cancer patients. Of note, the predictive model established in this study incorporates less lncRNA to increase its effectiveness for clinical application compared to the models from previously published studies.

Besides, there were some limitations in this study that should be addressed in future experiments. First, the immune-related lncRNA prediction models were established relying solely on the TCGA database. *In vivo* and *in vitro* experiments were not performed to verify the role of the models in liver cancer, therefore, subsequent experiments are needed to validate the reliability of results. Second, due to the limited lncRNA chip of liver cancer in the GEO database, we did not identify the corresponding probes for the two lncRNAs, thus GEO data was not used for verification.

Conclusively, through a comprehensive analysis of the TCGA database, this study identified an immune-related lncRNA signature to predict the prognosis of patients with liver cancer. The 2-lncRNA signature exhibited satisfactory robustness in the training group, testing group, and the entire dataset. Therefore, a nomogram model constructed based on the 2-lncRNA signature risk score can accurately predict the prognosis of HCC patients. This may provide a potential molecular marker for prognosis in HCC patients.

## Data Availability Statement

The data used to support the results of this study can be obtained from The Cancer Genome Atlas (TCGA) (https://cancergenome.nih.gov/).

## Author Contributions

WK, XW, XZ, ZM, and YC: data curation and software. WC: supervision. WK and XZ: writing – original draft. XW and WC: writing – review and editing. All authors read and approved the final manuscript.

## Conflict of Interest

The authors declare that the research was conducted in the absence of any commercial or financial relationships that could be construed as a potential conflict of interest.

## Footnotes

1 https://cancergenome.nih.gov/

2 https://www.cbioportal.org/index.do

3 https://www.gencodegenes.org/human/release_29.html

4 http://software.broadinstitute.org/gsea/register.jsp

5 http://software.broadinstitute.org/gsea/index.jsp

## Supplementary Material

FIGURE S1 Univariate Cox regression analysis to screen for prognostic lncRNAs in the training group.

FIGURE S2 The LASSO regression analysis for further screening the immune-related lncRNAs. LASSO, Least absolute shrinkage and selection operator.

FIGURE S3 A detailed example was showing how nomograms are used.

FILE S1 The accession IDs of liver cancer and normal samples included.

FILE S2 Immune-related lncRNAs were identified using the correlation analysis (Correlation > 0.5, *P*-value < 0.01).

FILE S3 The accession IDs of those included in the predictive models.

FILE S4 The enriched pathways of the high-risk group by GSEA.

FILE S5 The enriched pathways of the low-risk group by GSEA.
