# Supplementary figures and images for: Development and Validation of an Immune-Related lncRNA Signature for Predicting the Prognosis of Hepatocellular Carcinoma
Source: Front Genet. 2020 Sep 4;11:1037. doi: 10.3389/fgene.2020.01037 (PMC7500314; doi:10.3389/fgene.2020.01037)

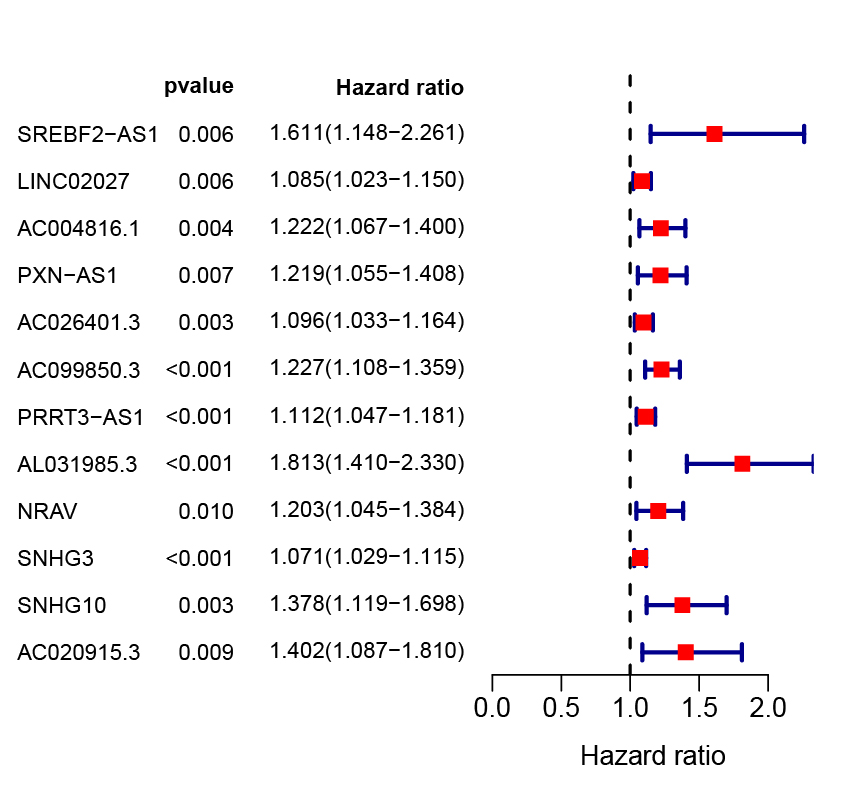

Supplement: FIGURE S1 — Univariate Cox regression analysis to screen for prognostic lncRNAs in the training group. [file Image_1.JPEG]

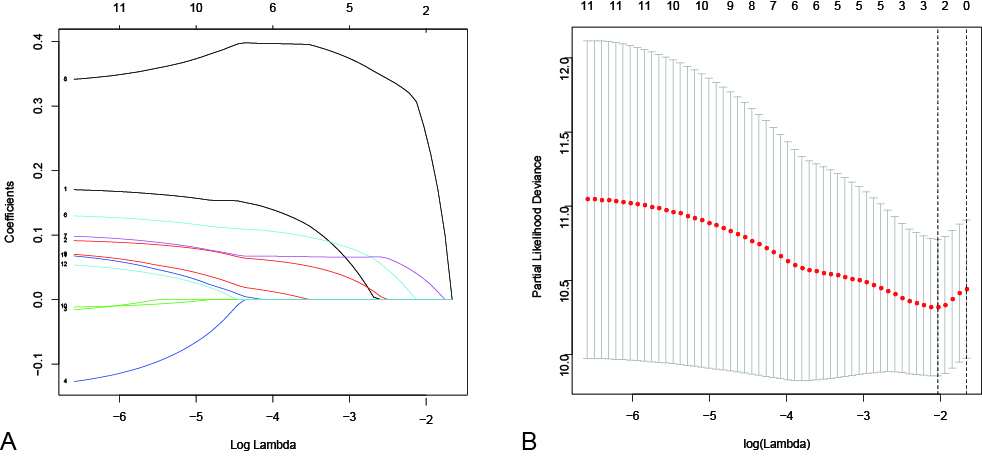

Supplement: FIGURE S2 — The LASSO regression analysis for further screening the immune-related lncRNAs. LASSO, Least absolute shrinkage and selection operator. [file Image_2.JPEG]

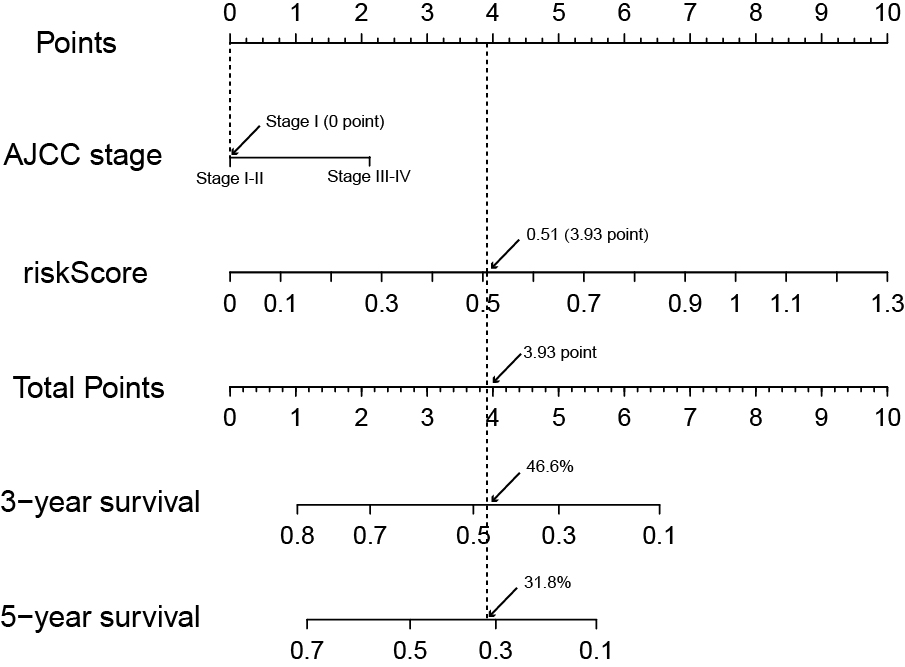

Supplement: FIGURE S3 — A detailed example was showing how nomograms are used. [file Image_3.JPEG]
